# Non-Inferiority of a Group LiFE Version Compared to the Original, Individual LiFE to Prevent Falls

**DOI:** 10.1093/geroni/igab046.1770

**Published:** 2021-12-17

**Authors:** Carl-Philipp Jansen, Corinna Nerz, Sarah Labudek, Sophie Gottschalk, Judith Dams, Jochen Klenk, Clemens Becker, Michael Schwenk

**Affiliations:** 1 Robert-Bosch-Hospital Stuttgart, Karlsruhe, Baden-Wurttemberg, Germany; 2 Robert-Bosch-Hospital, Stuttgart, Baden-Wurttemberg, Germany; 3 Heidelberg University, Heidelberg, Baden-Wurttemberg, Germany; 4 University Medical Center Hamburg-Eppendorf, Hamburg, Hamburg, Germany; 5 IB University of Applied Health and Social Sciences, Stuttgart, Baden-Wurttemberg, Germany

## Abstract

The ‘Lifestyle-integrated Functional Exercise’ (LiFE) program has been shown to reduce risk of falling via improvements in balance and strength while increasing physical activity in older adults. Its one-to-one delivery comes with considerable costs hampering large scale implementability. To potentially reduce costs, a group format (gLiFE) was developed and analyzed for its non-inferiority to LiFE in reducing activity-adjusted fall incidence after 6 months. Further, intervention costs and physical activity were analyzed. Older adults (70+ years) at risk of falling were included in this multi-centre, single-blinded, randomized non-inferiority trial. LiFE was delivered in nine intervention sessions to increase balance, strength, and physical activity, either in a group (gLiFE) or at the participant’s home (LiFE). 309 persons were randomized into gLiFE (n=153) and LiFE (n=156). Non-inferiority for activity-adjusted falls was inconclusive; the incidence risk ratio (IRR) of gLiFE was 1.350 (95% CI: 0.856; 2.128) at 6 months. Falls were largely reduced in both groups. Physical activity was superior in the gLiFE group (gLiFE +880 steps; CI 252, 1,509) which also had a cost advantage under study conditions as well as real world estimations. GLiFE was associated with lower intervention costs, making it a cost-efficient alternative to the individually delivered LiFE. The added value of gLiFE is the greater effect on physical activity, making it particularly attractive for large scale PA promotion in public health concepts. Depending on individual needs and preferences, both formats could be offered to individuals, with a greater focus on either fall prevention (LiFE) or physical activity promotion (gLiFE).

